# Carboxylate Adsorption on Rutile TiO_2_(100): Role of Coulomb Repulsion, Relaxation, and Steric Hindrance

**DOI:** 10.1021/acs.jpcc.1c00892

**Published:** 2021-06-17

**Authors:** Immad M. Nadeem, Laura Hargreaves, George T. Harrison, Hicham Idriss, Alexander L. Shluger, Geoff Thornton

**Affiliations:** † London Centre for Nanotechnology and Department of Chemistry, University College London, 20 Gordon Street, London WC1H 0AJ, United Kingdom; ‡ Diamond Light Source Ltd., Harwell Science and Innovation Campus, Didcot, Oxfordshire OX11 0DE, United Kingdom; § London Centre for Nanotechnology and Department of Physics and Astronomy, University College London, Gower Street, London WC1E 6BT, United Kingdom; ∥ Surface Science and Advanced Characterisation, Chemical Sciences Division, SABIC-CRD at KAUST, Thuwal, 23955 Saudi Arabia

## Abstract

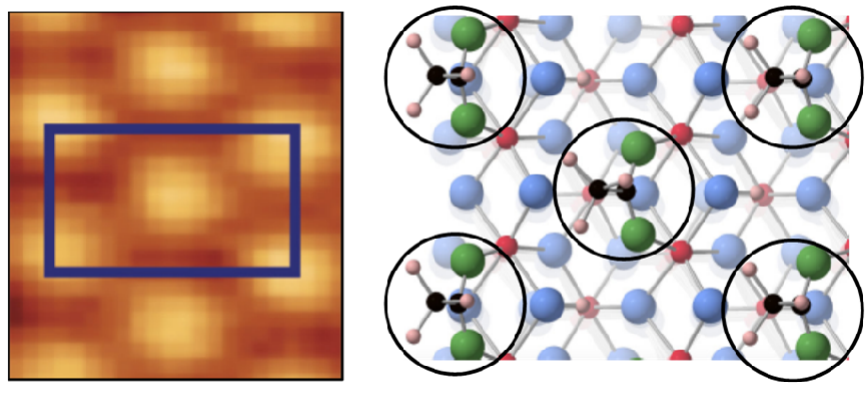

Understanding the adsorption and photoactivity of acetic acid and trimethyl acetic acid on TiO_2_ surfaces is important for improving the performance of photocatalysts and dye-sensitized solar cells. Here we present a structural study of adsorption on rutile TiO_2_(100)-1 × 1 and -1 × 3 using Scanning Tunnelling Microscopy and Density Functional Theory calculations. Exposure of both terminations to acetic acid gives rise to a ×2 periodicity in the [001] direction (i.e., along Ti rows), with a majority ordered c(2 × 2) phase in the case of the 1 × 1 termination. The DFT calculations suggest that the preference of c(2 × 2) over the 2 × 1 periodicity found for TiO_2_(110)-1 × 1 can be attributed to an increase in interadsorbate Coulomb repulsion. Exposure of TiO_2_(100)-1 × 1 and -1 × 3 to trimethyl acetic acid gives rise to largely disordered structures due to steric effects, with quasi-order occurring in small areas and near step edges where these effects are reduced.

## Introduction

Since Honda and Fujishima^1^ first demonstrated the photoelectrocatalytic capability of TiO_2_, the material has been widely investigated as a heterogeneous catalyst.^2,3^ The interactions of small organic molecules with rutile^4−6^ and anatase^7−9^ TiO_2_ surfaces have been the focus of numerous investigations.^10^ These act as model systems to elucidate the surface photoactivity associated with applications such as the photocatalytic degradation of organic pollutants.^6,11−15^ Carboxylate adsorption on TiO_2_ has also received much attention connected with dye adsorption on surfaces.^15−17^ In dye-sensitized solar cells (DSSC) the dye is typically bound to TiO_2_ nanoparticles via one or more carboxylate species. These oxygenates are preferred because the adsorption energy increases with the number of available oxygen atoms. More recently it has been proposed that TiO_2_ preferentially adsorbs atmospheric carboxylic acid species in preference to other, more abundant, adsorbate species.^18^ There are several factors that add complexity to the adsorption process, including dissociative versus molecular adsorption, the geometric (structural) effect of the underlying substrate, as well as the ligand effect. They significantly affect surface order and the associated coverage. These aspects motivate the current study, which investigates the face dependence of carboxylate adsorption.

Exposure of rutile TiO_2_(110)-1 × 1^4,5,19−22^ to acetic acid gives rise to a 2 × 1 acetate overlayer at saturation coverage. This results from dissociative adsorption with both oxygens binding to two adjacent surface Ti atoms, with the proton thought to bond to the adjacent surface O atom as a bridging OH species.^5,16,20^ A minority acetate species is thought to bond to oxygen vacancies or bridging OH species in a perpendicular orientation.^16^ Noncontact atomic force microscopy (NC-AFM)^22^ and scanning tunneling microscopy (STM)^4,5,21^ images of the 2 × 1 overlayers show domains that contain a few hundred adsorbates. Tip effects cause acetate molecules at the domain boundaries to move along the [001] direction.^22^ The stability of the 2 × 1 coverage has been attributed to attraction of acetate molecules across the [110] rows as well as to bridging OH species.^22^ Similarly, trimethyl acetic acid bonds dissociatively on TiO_2_(110)-1 × 1 forming a 2 × 1 overlayer at saturation coverage.^6,11,12^ Dissociatively adsorbed acetic acid molecules have also been reported to give rise to a 2 × 1 overlayer on TiO_2_(110)-1 × 1 at the solid/liquid interface.^4,20^

In parallel, DFT studies^23−25^ have also found that the 2 × 1 arrangement is the lowest energy configuration of small carboxylates on TiO_2_(110)-1 × 1. The calculations find that the coadsorption of H stabilizes the 2 × 1 coverage of formate by 0.02 eV per adsorbate compared with a c(2 × 2) periodicity.^24,25^ Moreover, the surface relaxations induced by the adsorption of acetate in the 2 × 1 arrangement maintain bond symmetries for surface 6-fold Ti cations. In contrast, these cations are destabilized by symmetry breaking in a c(2 × 2) adsorbate arrangement. This results in a total energy difference of 0.09 and 0.13 eV for supercell sizes of 4 × 2 and 6 × 2, respectively.^23^ Previous DFT studies of TiO_2_(100)-1 × 1 find dissociative bridging bidentate to be the lowest energy structure.^26^ In contrast, catechol (C_6_H_6_O_2_, which is an aromatic ring with two adjacent hydroxyls) is found to adsorb in a monodentate configuration on a stoichiometric surface and dissociative bidentate on an oxygen deficient surface.^27^

As for other TiO_2_ surfaces, exposure of rutile TiO_2_(011)-2 × 1 to acetic acid results in 1D clusters predicted by DFT to be a combination of molecular monodentate and dissociative bidentate adsorption.^5^ On anatase TiO_2_(101)-1 × 1,^7^ acetic acid adsorption at room temperature does not display long-range order. In contrast, adsorption at 420 K leads to a partially ordered 2 × 1 overlayer corresponding to dissociative bidentate adsorption. Exposure of anatase TiO_2_(001)-1 × 4 thin films to acetic acid forms a 4 × 2 overlayer of acetate.^9^

Rutile TiO_2_ terminations exhibit a surface stability order of (110) > (100) > (011),^28,29^ with the (110) and (011) terminations being studied more extensively.^15,16^ This probably arises from the numerous and complex possible terminations attributed to TiO_2_(100), that is, 1 × 1,^30−32^ 1 × 2,^33^ 1 × 3,^31,34,35^ 1 × 5,^36^ 1 × 7,^37^ and c(2 × 2).^38^ The 1 × 3 termination exists as a “microfacet” (1 × 3^MF^) termination, as well as the intermediate 1 × 3^α^ and 1 × 3^β^ structures^31,39^ that form as a transition from 1 × 1 to 1 × 3^MF^. The microfacet structure is understood to increase its stability through its (110) faceting.^31^ In this paper we compare carboxylate adsorption on the 1 × 1 termination and the 1 × 3^MF^ terminations of the TiO_2_(100) surface. We find that Coulomb repulsion between adsorbates, relaxation, the presence of hydroxyls, and steric effects all play a role in determining the adsorption energies.

## Experimental and Computational Details

A rutile TiO_2_(100) single crystal (*Pi-Kem*) was mounted on a Ta plate with Ta clips and degassed in ultrahigh vacuum (UHV). Surfaces were prepared with cycles of Ar^+^ sputtering (P_Ar_ = 8 × 10^–5^ mbar, 1 keV, 10 μA cm^–2^, 10 min) and annealing (TiO_2_(100)-1 × 1: ≤ 973 K; TiO_2_(100)-1 × 3^MF^: ≤ 1273 K). The UHV preparations of rutile TiO_2_(100)-1 × 1 and -1 × 3^MF^ differ in the anneal temperature employed, with a higher anneal temperature leading to the reduced 1 × 3^MF^ reconstruction. A similar behavior is observed for TiO_2_(110), where a higher anneal temperature results in the formation of the reduced 2 × 1 reconstruction.^15,16^

Low energy electron diffraction (LEED) and Auger electron spectroscopy (AES) were used to ensure an ordered and contaminant-free surface (below the detection limits of AES) prior to STM measurements. The latter were performed at room temperature with an Omicron AFM/STM instrument with a base pressure of ∼1 × 10^–10^ mbar. STM was performed in constant current mode with electrochemically etched tungsten tips that were degassed in UHV and conditioned during scanning with voltage pulses and high bias scans (up to ±10 V). Surfaces were imaged by tunneling into empty states. It was possible to image the overlayers at a sample bias of +1 and +1.6 V. The images shown below were recorded at +1.6 V.

Acetic acid and trimethyl acetic acid (Sigma-Aldrich) were dosed into the UHV system via a high precision leak valve after degassing with several freeze–pump–thaw cycles. In this paper exposures are quoted in Langmuir, where 1 L = 1.33 × 10^–6^ mbar s. The exposure was around 1.5 L in all cases. This level of exposure was found to give a saturated 2 × 1 coverage of acetate on TiO_2_(110) in the same chamber. Surface coverages are given with respect to the number of surface unit cells of TiO_2_(100)-1 × 1. Acetic acid and trimethyl acetic acid were dosed onto the room temperature surface at a partial pressure of 1 × 10^–8^ mbar.

Density functional theory (DFT) calculations were employed to study the adsorption of acetate on rutile TiO_2_(100)-1 × 1, as well as TiO_2_(110)-1 × 1 for comparison. We use the CP2K^40^ code, which implements a mixed Gaussian and plane wave basis-set (GPW). A triple-ζ basis set was used for Ti, O, C, and H in combination with the Goedecker–Teter–Hutter (GTH) pseudopotentials.^41^ The planewave cutoff was 600 Ry, with the electronic structure and residual forces being converged to 10^–6^ a.u. and 0.1 eV nm^–1^, respectively. In calculations presented here, we used the HSE06 functional^42^ with 25% Hartree–Fock (HF) exchange and the ω parameter set to 0.11 Bohr^–1^. HSE06 yields the band gap of TiO_2_^43,44^ as well as the ionization potentials and vertical excitation energies of the acetate molecule in agreement with experimental data. CP2K employs the auxiliary density matrix method (ADMM),^45^ where a reduced basis set is used for Hartree–Fock exchange calculations to reduce the computational cost. All calculations were carried out at the Γ point.

Bulk rutile TiO_2_ cell optimization calculations yield lattice vectors; *a* = 0.459 nm, *c* = 0.295 nm, and a band gap of 3.31 eV. The TiO_2_(100)-1 × 1 and (110)-1 × 1 terminations were modeled by a (2 × 4) surface slab in the *x* and *z* directions with the surface normal parallel to the *y* direction. Surface properties are found to depend on the number of layers in the slab model.^46^ The number of layers in the slab was converged to 10 and 8 for TiO_2_(100)-1 × 1 and (110)-1 × 1, respectively, and the vacuum gap was 1.5 nm at either side of the surface slab.

Calculations of acetate adsorption were performed on one side of a stoichiometric surface. For each acetate in the system, a hydroxyl group was positioned nearby to mimic the expected dissociation of acetate. As adsorption was performed on one side of the slab, surface dipole corrections were applied to the asymmetric system. 2D boundary conditions were used in the *x* and *z* directions. All calculations were performed at 0 K. The inclusion of dispersion forces had a negligible effect on adsorption trends, in agreement with earlier work.^23−25^ Adsorption energies, *E*_ads_, per acetate molecule were calculated as
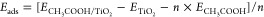
where *E*_CH_3_COOH/TiO_2__ is the total energy of the adsorbate system, *E*_TiO_2__ is the total energy of a pristine slab, *E*_CH_3_COOH_ is the total energy of the acetic acid molecule in the gas phase, and *n* is the number of acetate molecules.

The net total energy difference between the two adsorption configurations, *E*_net_, is calculated as
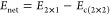
where *E*_2×1_ and *E*_c(2×2)_ are the total energies of the 2 × 1 and c(2 × 2) coverage, respectively. The distortion energy, *E*_dist_, of the surface per acetate molecule is calculated as
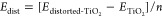
where *E*_distorted-TiO_2__ is the energy of the distorted surface slab without any adsorbates.

## Results and Discussion

### Acetate Adsorption on TiO_2_(100)

**Figure 1:**
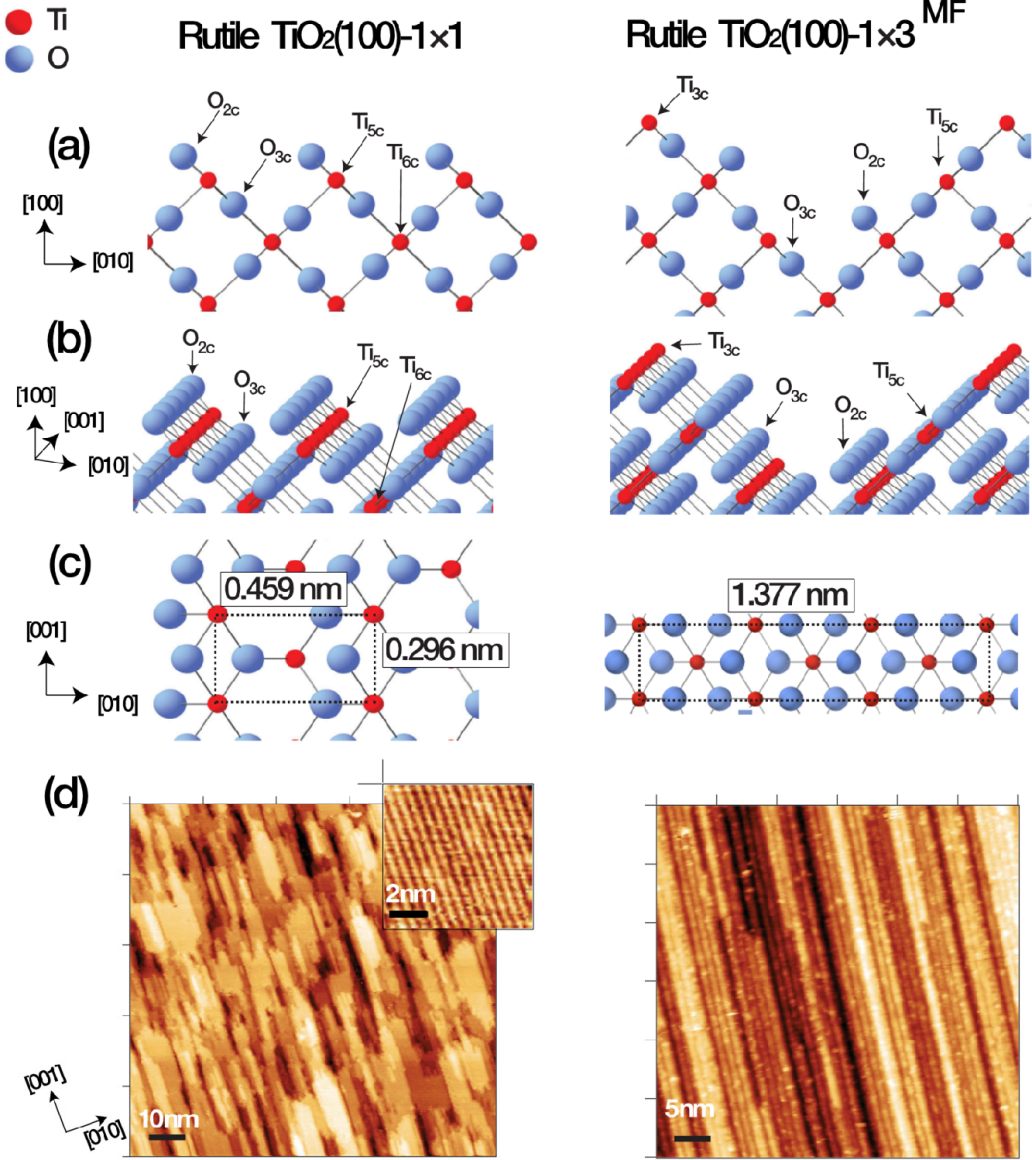
(a) Side, (b) oblique, and (c) on-top views, respectively, depicting TiO_2_(100)-1 × 1 (left) and -1 × 3_MF_ (right) with surface atoms Ti_6c_, Ti_5c_, Ti_3c_, O_3c_, and O_2c_ labeled. (d) Left: 100 × 100 nm^2^ STM image of TiO_2_(100)-1 × 1 (*V*_s_ = +1.6 V, *I*_t_ = 0.1 nA) with inset (8 × 8 nm^2^) containing “atomically resolved” features. Right: 50 × 50 nm^2^ STM image of TiO_2_ (100)-1 × 3^MF^ (*V*_s_ = +1.6 V, *I*_t_ = 0.1 nA).

Figure 1 contains ball and stick models and STM images of the two TiO_2_(100) terminations investigated in this work. STM images of the 1 × 1 surface contain bright and dark rows with a separation of 0.459 nm in the [010] direction and 0.296 nm in the [001] direction. Images of the 1 × 3^MF^ surface contain thick strands running along the [001] direction with a spacing of 1.377 nm in the [010] direction and 0.296 nm in the [001] direction.

### STM Studies of Acetate Adsorption on TiO_2_(100)-1 × 1

**Figure 2:**
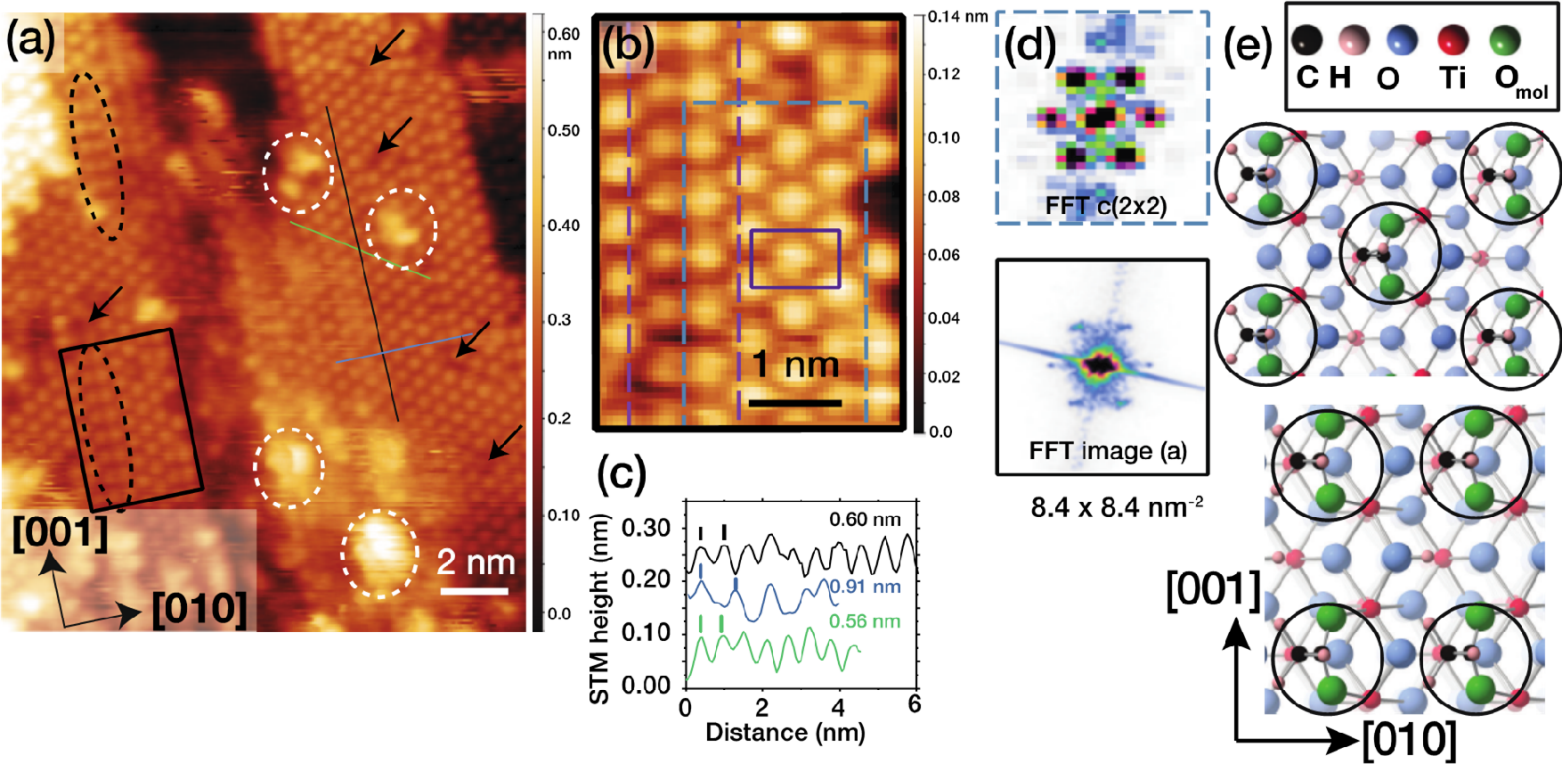
(a) 15.7 × 18.4 nm^2^ STM image of TiO_2_(100)-1 × 1 (*V*_s_ = +1.6 V, *I*_t_ = 0.1 nA) after a 1.5 L exposure to acetic acid. Line profiles in (c) were obtained from the black line in the [001] direction, the blue line along [010] and the green line along the diagonal of the surface unit cell. Black dashed ovals contain areas of 2 × 1 symmetry, white dashed ovals identify features arising from adventitious adsorption, arrows indicate defects in the adlayer, and the black rectangle is expanded in (b). (b) An area of c(2 × 2) symmetry is identified with the light blue dashed box, with the purple rectangle showing the unit cell. The purple dashed box identifies a (2 × 1) domain. (c) Line profiles from the image in (a), with average spacings indicated. (d) FFT of the c(2 × 2) domain in the green dashed box in (b) and the FFT of image (a). (e) Models of the c(2 × 2) (top) and (2 × 1) (bottom) overlayers. The average minimum distance between adsorbates in (a) is 0.50 ± 0.03 nm.

**Figure 3:**
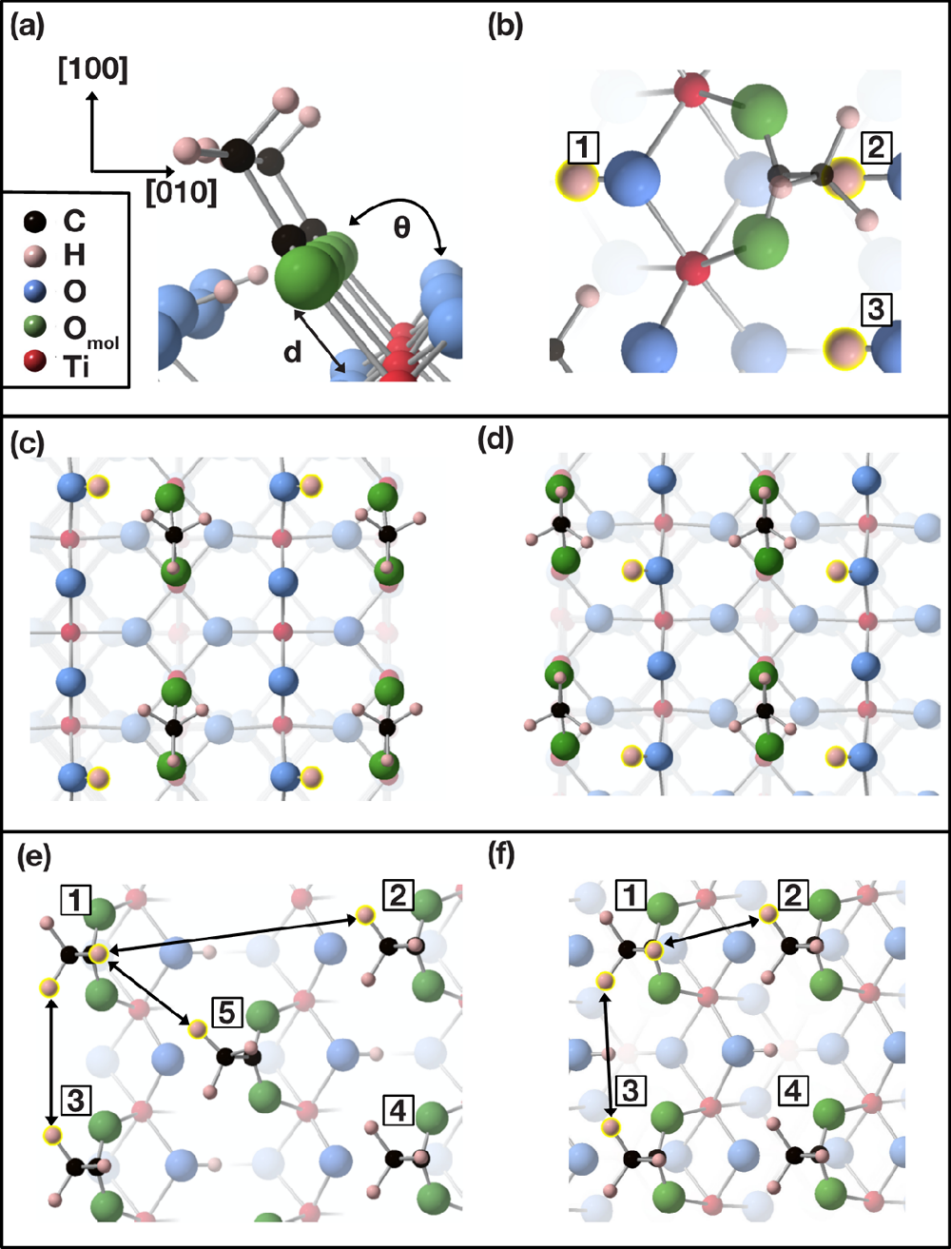
Schematic diagrams of acetate adsorbed in the c(2 × 2) and 2 × 1 overlayers on the TiO_2_(100)-1 × 1 and (110)-1 × 1 surfaces. (a) Acetate adsorption on the (100)-1 × 1 surface in the bridging bidentate adsorption. Labels *d* and Θ describe the bond length and angle of adsorption between acetate and the surface, respectively. Their values are listed in Table 1. (b) Positions 1, 2, and 3 of the hydroxyl group on the (100)-1 × 1 surface with respect to the acetate. (c), (d) Possible hydroxyl positions on the (110)-1 × 1 surface. (e), (f) Nearest neighbor distances between acetate molecules in the TiO_2_(100)-c(2 × 2) and 2 × 1 overlayers, respectively. Acetate molecules are labeled 1–5 and their corresponding distances are listed in Table 3. Distances are taken between the hydrogens of the methyl groups of acetate.

Figure 2a shows a STM image recorded following exposure to 1.5 L acetic acid. The adsorbates form ordered overlayer domains on TiO_2_(100)-1 × 1 with a spacing in the [001] direction of 0.58 ± 0.02 nm and a spacing of 0.45 ± 0.01 nm in the [010] direction. Two types of domain are observed. In the majority, adsorbates are out of phase along [001] in adjacent [001] direction rows, giving a c(2 × 2) periodicity (see Figure 2b). This periodicity is confirmed by line scans (Figure 2c), where the adsorbate separations are those expected on the basis of the unit cell dimensions, as well as fast Fourier transforms (FFT; Figure 2d) of the images in Figures 2a,b. This contrasts with the (110) termination, where the majority domain has a 2 × 1 periodicity.^4,5,19−22^ This is the periodicity adopted by the minority domain type on TiO_2_(100)-1 × 1 (see Figure 2a,b). These line defects, as well as point defects in the overlayer can occur due to underlying substrate defects on the as-prepared surface. It is also possible that they could be associated with OH species resulting from acid deprotonation.^5,16^ The measured acetate coverage of 0.37 ± 0.02 ML is only slightly lower than expected for a perfect overlayer (0.5 ML), which arises from the presence of these defects. Consistent with this, the average minimum distance between adsorbates is found to be 0.50 ± 0.03 nm. This is close to the nearest neighbor distance in the c(2 × 2) overlayer, 0.55 nm. As expected from previous studies^10,15,16^ of carboxylates on TiO_2_, acetate appears strongly bound to the TiO_2_(100)-1 × 1 substrate. Successive STM scans over a few hours result in no changes to the overlayer structure and STM tip pulsing of the overlayer indicated little or no change at tip pulses up to +6 V. This suggests that acetic acid is dissociatively adsorbed, with acetate bonded to adjacent Ti_5c_ in a bidentate configuration as found for other TiO_2_ surfaces exposed to carboxylic acids.^4,7,20^ Models depicting this geometry are shown in Figures 2e and 3a.

### DFT Calculations of Acetate on TiO_2_(100)-1 × 1 and (110)-1 × 1

To understand the origins of stability of the c(2 × 2) domain on the TiO_2_(100)-1 × 1 surface, we performed DFT calculations of the adsorption of acetate at saturation coverage in both c(2 × 2) and 2 × 1 arrangements. Since a large number of both experimental and theoretical studies exist for acetate and formate adsorption on TiO_2_(110)-1 × 1, we also performed calculations for acetate on this substrate for comparison. Moreover, we explore the role of nearby hydroxyl groups since several DFT studies^47−51^ have predicted that the relative stability of dissociated adsorbate geometries depends on these coadsorbates. The positions of the hydroxyl group on TiO_2_(100)-1 × 1 (see Figure 3b) were calculated for acetate at saturation coverage with the HSE06 functional. Under periodic boundary conditions, positions 1 and 2 are equivalent for the 2 × 1 coverage and positions 1 and 3 are equivalent for the c(2 × 2) coverage. It was found that position 3 is the lowest energy configuration for both the c(2 × 2) and 2 × 1 overlayers by 0.35 and 0.41 eV, respectively. On the TiO_2_ (110)-1 × 1 surface, the O_b_ atoms and Ti_5c_ atoms are in line along the [110] direction. Therefore, there are two positions of the hydroxyl group with respect to a single acetate molecule that are the same distance from the oxygens of acetate. At saturation coverage, the energy is the lowest by 0.03 eV when an acetate molecule and a neighboring hydroxyl group are in the position shown in Figure 3c for both overlayers. In comparison, a previous STM study reports that hydroxyl groups are evenly spaced after the removal of the acetate adsorbate (Figure 3d),^52^ although this may be facilitated by the energy imparted to the surface.

Table 1 lists the adsorption properties of acetate at saturation coverage in the 2 × 1 and c(2 × 2) arrangements on TiO_2_(100)-1 × 1 and (110)-1 × 1. Smaller adsorption energies per acetate molecule were found when they formed the 2 × 1 coverage in comparison to the c(2 × 2) coverage by 0.12 eV on the (100)-1 × 1 surface. This is consistent with the experimental results, which show a dominance of c(2 × 2) periodicity. In contrast, for TiO_2_(110)-1 × 1, the difference in adsorption energies per acetate between the two coverages was found to be 0.02 eV. The total energetic difference between the 2 × 1 and c(2 × 2) overlayers on TiO_2_ (110) is predicted to be 0.06 eV. This is consistent with the results of the previous calculations, where the net energetic difference *E*_net_ was reported to be 0.09 eV for a 4 × 2 × 5 surface slab and 0.13 eV for a 6 × 2 × 5 surface slab.^23^

**Table 1:** Optimized Energy and Geometry Parameters for Acetate Adsorption on the Rutile TiO_2_ (100)-1 × 1 and (110)-1 × 1 Terminations^a^

acetate periodicity	rutile TiO_2_ substrate	*E*_a_ (eV)	*E*_dist_ (eV)	*d*(Ti–O_mol_) (nm)	Θ (°)
2 × 1	(100)-1 × 1	–1.24	1.35	0.206–0.207	86.8
	(110)-1 × 1	–1.50	1.94	0.205–0.211	90.0
c(2 × 2)	(100)-1 × 1	–1.36	1.35	0.202–0.203	86.2
	(110)-1 × 1	–1.48	1.94	0.205–0.211	90.0

a *E*_a_ is adsorption energy; *E*_dist_ is the distortion energy of the surface; Ti–O_mol_ is the distance between the Ti_5c_ and O acetate atom, labelled (d) in Figure 3a; the last column describes the angle of adsorption, Θ. This is illustrated for TiO_2_(100)-1 × 1 in Figure 3a.

**Figure 4:**
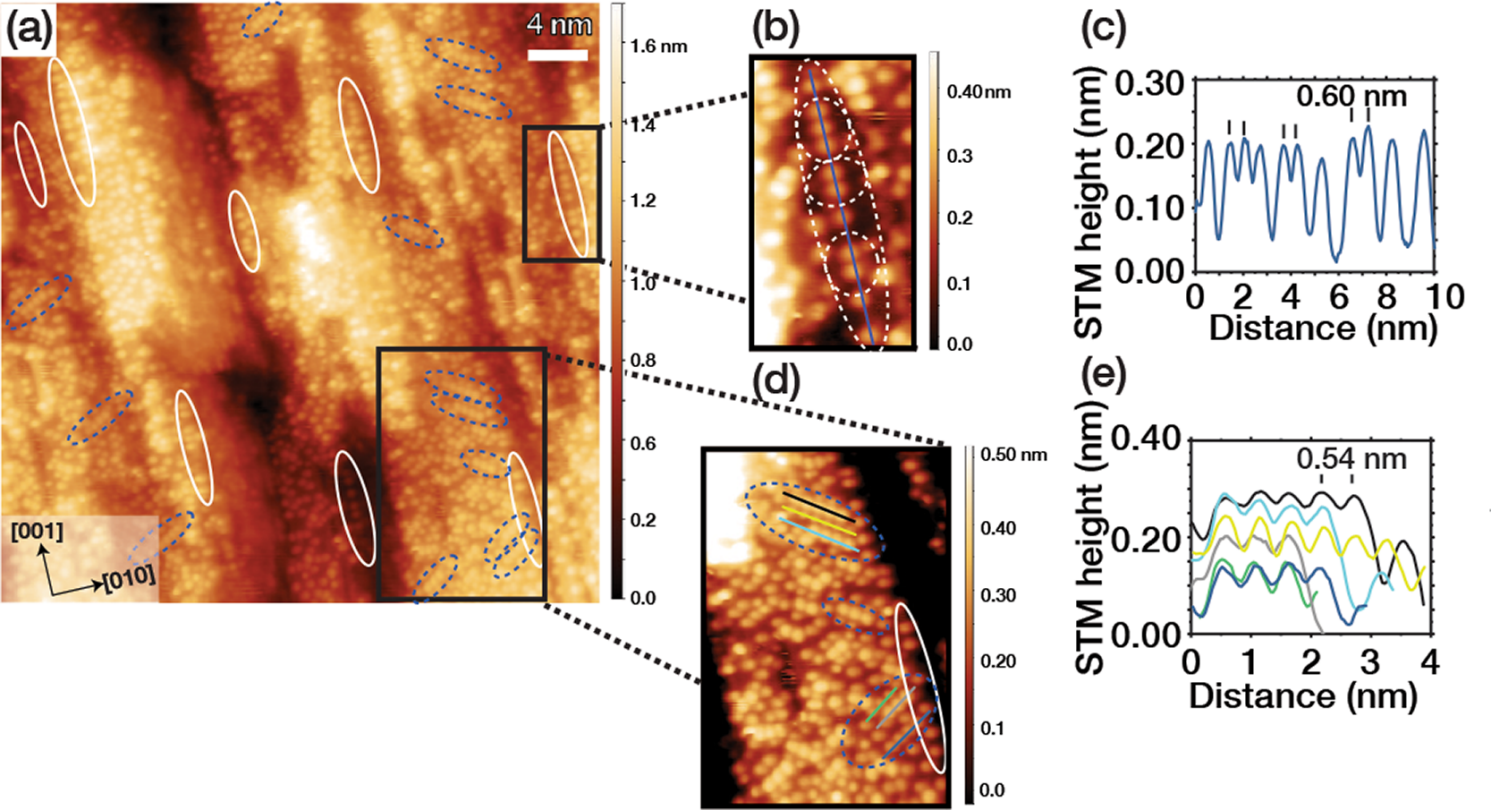
STM images of TiO_2_(100)-1 × 1 (*V*_s_ = +1.6 V, *I*_t_ = 0.1 nA) after exposure of TiO_2_(100)-1 × 1 to 1.5 L trimethyl acetic acid. (a) 40 × 40 nm^2^, solid white ovals depict quasi-ordering of trimethyl acetate along [001]-direction step-edge rows. Blue dashed ovals depict ordering along diagonal directions. (b) 4.8 × 8.4 nm^2^ zoom of a step edge in which white-dashed ovals depict areas of ×2 periodicity of adsorbates. (c) A line profile of the [001] direction blue line. This points to a separation between adsorbates of about 0.60 nm. (d) 11 × 17 nm^2^ zoom of part of the image in (a). Blue-dashed ovals indicate local ordering along the diagonal directions. (e) Line profiles from the image in (d), evidencing an average separation of 0.54 nm. The average minimum distance between adsorbates in (a) is 0.57 ± 0.15 nm.

Shorter adsorbate–surface bond lengths were found when acetate is in the c(2 × 2) overlayer on TiO_2_(100)-1 × 1. Acetate adsorbs perpendicular to the (110)-1 × 1 surface, whereas due to the sawtooth topology of TiO_2_(100)1 × 1, seen in Figures 1a and 4, the steric hindrance from the bridging oxygens prevents acetate from adsorbing perpendicular to the facet. The adsorption distance between acetate and the Ti_5c_ atoms on TiO_2_(110)-1 × 1 did not depend on the overlayer periodicity.

The partial charges of the anions and cations that are involved in adsorption calculated by Bader population analysis^53^ are listed in Table 2. At both coverages, a larger net negative charge is attributed to oxygens of the acetate molecules in comparison to the bridging oxygens of the surface. Hydrogen atoms of the hydroxyl groups are predicted to have a net positive charge. These results are consistent with the notion that acetate is deprotonated, leading to a net negative charge on the acetate molecules.

**Table 2:** Partial Charges for Acetate Atoms and Surface Atoms Involved in Adsorption for the c(2 × 2) and 2 × 1 Overlayers on TiO_2_(100)-1 × 1 and (110)-1 × 1^a^

		partial charge/*e*	
overlayer periodicity	entity	(100)	(110)
2 × 1	O_mol_	–1.74/–1.79	–1.77
	Ti_5c_	2.37	2.38/2.39
	O_b_	–1.25	–1.24
	H	0.63	0.62
c(2 × 2)	O_mol_	–1.72/–1.77	–1.77^b^
	Ti_5c_	2.36/2.37	2.37/2.39
	O_b_	–1.25	–1.24
	H	0.64	0.62

a O_mol_ and Ti_5c_ are the partial charges of the acetate oxygen atoms and the titanium atoms directly below acetate molecules. O_b_ and H are the partial charges for the oxygen and hydrogen of the hydroxyl group.

b Average value provided for oxygen adsorbates.

Table 3 lists the distances between acetate molecules and their neighbors, which are described in Figure 3e,f. Packing in the c(2 × 2) overlayer is less dense than in the 2 × 1 coverage on both TiO_2_(100)-1 × 1 and (110)-1 × 1. Furthermore, the (110)-1 × 1 surface unit cell is larger than that for the (100)-1 × 1 surface. The adsorbate coverage is therefore less dense leading to smaller intermolecular interactions compared with the (100)-1 × 1 surface. The nearest neighbor distance along the [001] direction (between atoms 1–3, Figure 3e,f) is smaller by 0.010 nm on the (110)-1 × 1 surface for the 2 × 1 overlayer in comparison to the c(2 × 2) overlayer; this results from acetate molecules twisting to increase the interaction with the proton. The distance between the proton and acetate oxygen is found to be 0.222–0.225 nm and 0.226–0.227 nm for the 2 × 1 and c(2 × 2) overlayers, respectively.

**Table 3:** Distances between Acetate Molecules for c(2 × 2) and 2 × 1 Overlayers on TiO_2_ (100)-1 × 1 and (110)-1 × 1^a^

surface		*d*(1–2) (nm)	*d*(1–3) (nm)	*d*(1–5) (nm)
c(2 × 2)	(100)-1 × 1	0.782	0.412	0.388
	(110)-1 × 1	1.122	0.427	0.520
				*d*(1–4) (nm)
2 × 1	(100)-1 × 1	0.338	0.411	0.597
	(110)-1 × 1	0.472	0.437	0.708

a The acetate molecules are numbered in the unit cells in Figure 3e,f. The distances are taken between hydrogen of the methyl groups as calculated by DFT.

On TiO_2_(110)-1 × 1 we find that acetate molecules adsorbed along the same Ti_5c_ row (*d*(1–3)) are closer together in the c(2 × 2) overlayer than in the 2 × 1 overlayer. In the latter configuration, there is increased repulsion between acetate molecules as they face each other either side of a O_b_ rows (*d*(1–2)). This repulsion may encourage acetate molecules to rotate in a way that both helps to maximize the electrostatic attraction between the hydroxyl group and acetate as well as reducing electrostatic repulsion between the acetate molecules along the same Ti_5c_ row.

Atomic displacements on the surface occur to accommodate the bridging bidentate geometry of acetate. Rumpling in the first and second layers has been observed by LEED-IV for formate on TiO_2_(110)^54^ and by DFT calculations for acetate on TiO_2_(110).^20^ A previous DFT study suggested that the expansions and contractions of surface bonds caused by adsorption could be one of the important factors for the preferred 2 × 1 arrangement of acetate on the (110)-1 × 1 surface.^23^ In this work, we considered the energetic contribution from the surface relaxations, which we define as the distortion energy. This energy is significant and describes the total energetic difference per adsorbate between a clean surface slab at the coordinates of adsorption and a clean perfect slab. There is negligible difference between the distortion energies shown in Table 1 for both the c(2 × 2) and 2 × 1 coverage. This suggests that the energetic contribution of relaxation induced by adsorption is not sensitive to the arrangement of acetate molecules. However, the relaxation associated with the hydroxyl groups on the (100) surface is quite significant. The hydrogen pulls the O_b_ atom along the [010] direction and downward toward the O_3c_ atom (see Figure 1a). This relaxation stretches the Ti_5c_ and O_b_ bond where the hydroxyl is present by 9.29% (see Figure 3b, position 1 for c(2 × 2) coverage and position 2 for 2 × 1 coverage). Where no hydroxyl is present, this length contracts by 0.87% and 0.87–1.37% for the c(2 × 2) and 2 × 1 overlayers, respectively. This is also demonstrated by the displacement of Ti_5c_ atoms. For the c(2 × 2) overlayer the hydroxyl group is bonded to the Ti_5c_ atoms that are interacting with acetate. These atoms are found to move away from each other by 4.07% and the neighboring distance to contract by 4.07%. In the 2 × 1 overlayer, hydroxyl groups are bonded to Ti_5c_ atoms that are not bound to acetate. They are found to increase their distance by 5.76%, and the neighboring distance to contract by 5.76%. For the TiO_2_(100)-1 × 1 surface, we propose that the repulsion between the negatively charged acetates results in the c(2 × 2) overlayer being more favorable than the 2 × 1 adlayer. This effect is more important on the TiO_2_(100)-1 × 1 surface as the distance between acetate molecules on neighboring rows is smaller than on the (110)-1 × 1 surface. There is no experimental evidence of a c(2 × 2) overlayer on the (110)-1 × 1 surface, suggesting that the origins of the predominance of the (2 × 1) overlayer should be explored in future theoretical work.

Finally, we note that excess electrons and hydrogen in the subsurface of TiO_2_(110) are known to arise from defects and hydrogen diffusion following H_2_ dissociation, respectively. The effect of excess electrons on the overlayer symmetry is not thought to be significant because the density of excess electrons is only 0.03 per surface unit cell based on that measured for the (110) surface.^55^ Moreover, a recent study evidences little modification of the surface polaron density on acetic acid dissociative adsorption.^56^ As for the influence of subsurface hydrogen, there are numerous studies that suggest that the protons remain at the surface in the form of bridging hydroxyls following dissociative adsorption of carboxylic acids. For instance, an SXRD study in conjunction with DFT showed that acetic acid dissociates on TiO_2_(110) to form a negatively charged carboxylate and a proton.^20^

### Trimethyl Acetate Adsorption on TiO_2_(100)-1 × 1

**Figure 5:**
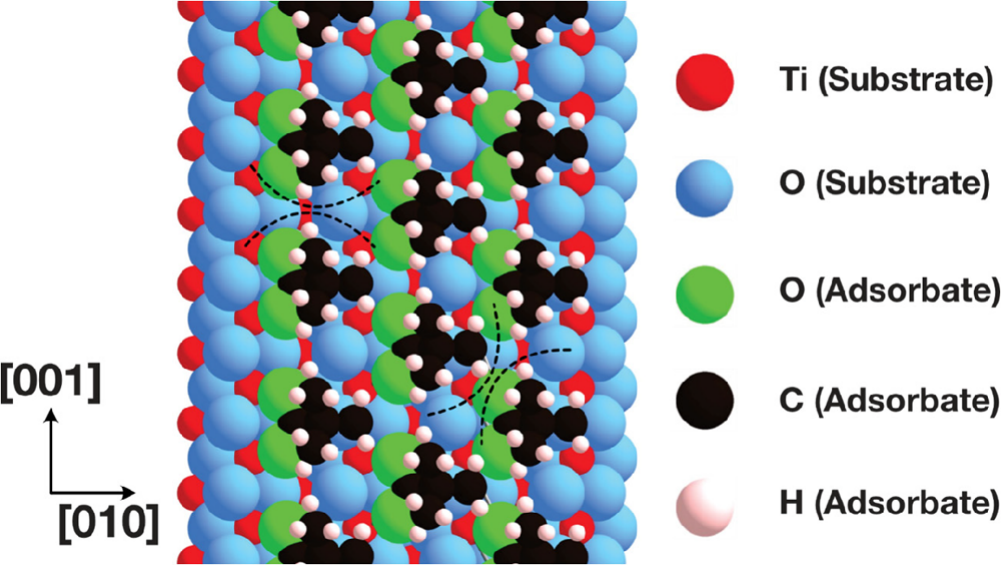
On-top model of trimethyl acetic acid binding to TiO_2_(100)-1 × 1 in a hypothetical c(2 × 2) arrangement. The dashed black curves highlight the steric hindrance effects associated with adjacent CH_3_ species that prevents the formation of an ordered overlayer. Covalent radii of the adsorbate C and H atoms are used in the models. Hydroxyl groups are omitted for clarity.

Figure 4 shows STM images of TiO_2_(100)-1 × 1 after exposure to 1.5 L trimethyl acetic acid, which results in a coverage of 0.27 ± 0.04 ML. The average minimum distance between adsorbates, 0.57 ± 0.15 nm, suggesting that it may be possible to obtain a greater coverage by adsorption between existing domains. The disordered nature of the adsorbates is in contrast to that observed following exposure to acetic acid (see Figure 2). It also contrasts with the behavior of TiO_2_(110)-1 × 1, where exposure to both acetic acid and trimethyl acetic acid results in a 2 × 1 overlayer of the carboxylate. The disorder in the case of TiO_2_(100)-1 × 1 could arise from nondissociative adsorption, although this is unlikely based on the stability of the overlayer. Indeed, the overlayer is as robust as acetate to STM scanning and tip pulsing. A more likely explanation is that the far bulkier alkyl chain prevents formation of even the c(2 × 2) overlayer observed for acetate. Figure 5 shows a model of trimethyl acetate arranged on TiO_2_(100)-1 × 1 with a hypothetical c(2 × 2) periodicity, where the bond lengths of the carboxylate group are taken from crystallographic measurements of acetate on TiO_2_(110)-1 × 1.^20^ In Figure 5, the steric hindrance effects of the −CH_3_ groups are immediately evidenced given the proximity of the H atoms from adjacent adsorbate molecules. This is consistent with the ×2 ordering that is seen in some cases for the adsorbates along [001] step edge rows (Figure 4b,c), where this steric effect would be removed in the [010] direction. A limited number of short ordered linear arrays is also observed in the direction diagonal to the principal azimuths, at an angle of about 57° to the [001] direction. This corresponds to the nearest neighbor distance direction in the c(2 × 2) unit cell, consistent with the line profile separation. Although this is consistent with an attractive interaction, the rows are separated by disordered areas, which will reduce steric effects. The similarity of the adsorbate separation in the one-dimensional ordered areas to those of acetate on (100)-1 × 1 suggests that trimethyl acetic acid also dissociatively adsorbs to bidentate bond to adjacent Ti_5c_ atoms.

Given the arrangement of the C–C axis, strong interactions with the substrate atoms are also likely to prevent ordered adsorption. In comparison, the (CH_3_)_3_C–COO bond will lie along the surface normal on TiO_2_(110), with the H atoms being higher above the surface when compared to the angled carboxylate adsorption expected for TiO_2_(100)-1 × 1. Moreover, the distance of closest approach of −CH_3_ of adjacent adsorbates is 0.302 nm (along the Ti rows) and 0.359 nm (across the Ti rows).

### Acetate and Trimethyl Acetate Adsorption on TiO_2_(100)-1 × 3

**Figure 6:**
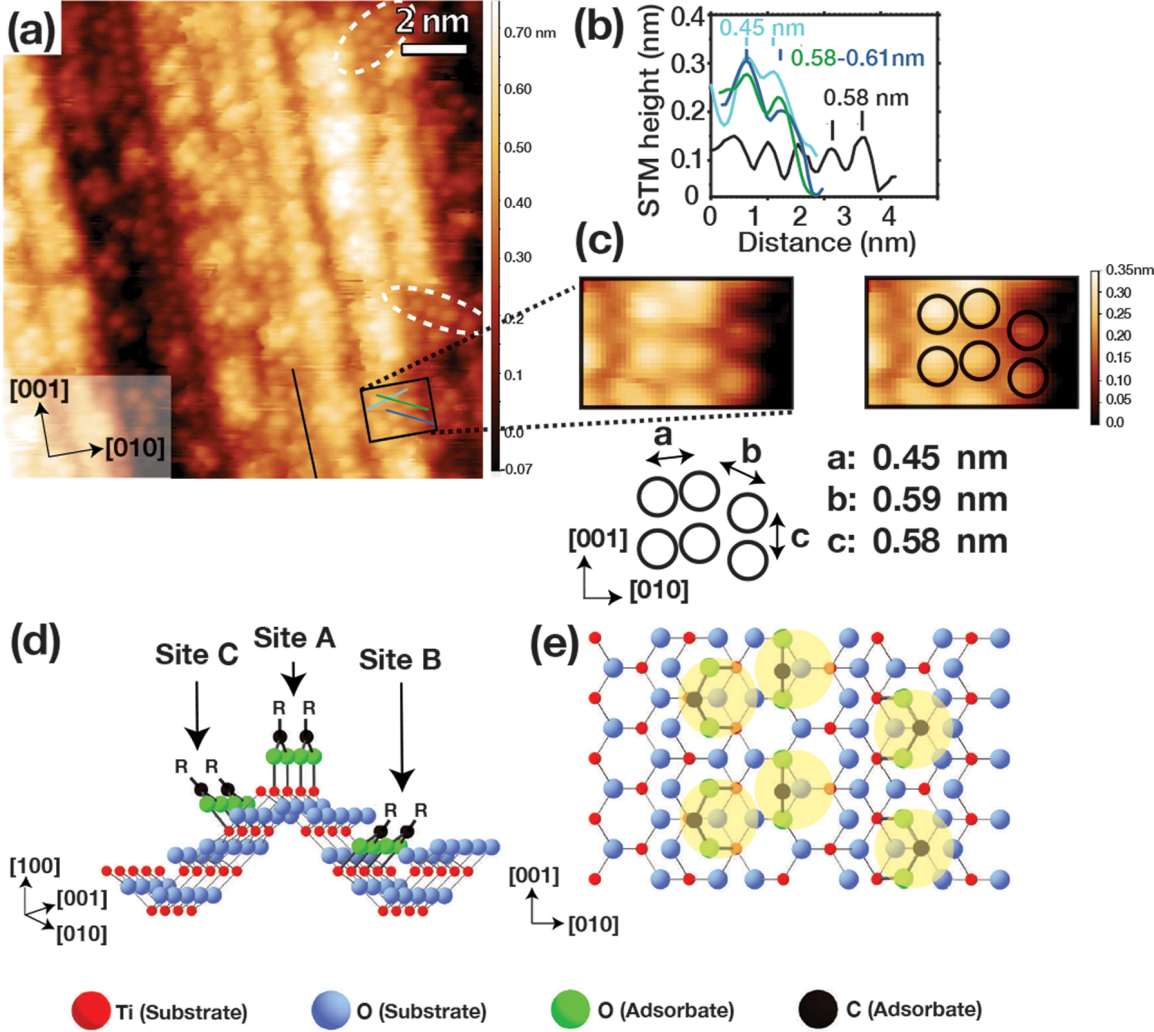
(a) 15 × 15 nm^2^ STM image of TiO_2_(100)-1 × 3 MF (*V*_s_ = +1.6 V, *I*_t_ = 0.1 nA) after exposure to 1.5 L acetic acid with white dashed ovals depicting ordering along the [001] and diagonal directions. (b) Line profiles from the image in (a), black along the [001] direction and blue, dark blue and green arranged to measure ordering among sites a, b and c shown in (c). (c) 2.6 × 1.8 nm^2^ zoom of part of the image in (a). Black circles are used to highlight the adsorbate positions in the right-hand image, with the distances obtained from (b) shown on the adsorbate arrangement. (d) Ball and stick model of TiO_2_(100)-1 × 3 MF with three potential carboxylate adsorption sites labeled. (e) Ball and stick model with yellow circles highlighting the acetate arrangement seen in (c). R represents CH_3_. The average minimum distance between adsorbates in (a) is 0.45 ± 0.07 nm.

Figure 6 shows the relevant structures and STM images of TiO_2_(100)-1 × 3^MF^ following exposure to 1.5 L acetic acid, which gives rise to an adsorbate coverage of 0.37 ± 0.02 ML. This overlayer is as robust to STM scanning and tip pulsing as acetate on the TiO_2_(100)-1 × 1 termination, which we take to indicate that the adsorbates observed on TiO_2_(100)-1 × 3^MF^ are also bidentate acetate. As is seen in Figure 6a,b, the acetate exhibits partial local order. The lack of order in the [010] direction can be attributed to the positioning of the three types of potential adsorption sites (see Figure 6c), such that adsorption at adjacent sites is unlikely because of steric effects.

Figure 6c,d gives a pictorial representation of an instance where acetate binds to sites A, B, and C. The distances here are consistent with the values obtained from the line profiles in Figure 6c. Although the 1 × 3 termination is expected to be more reactive than the 1 × 1 surface because it contains Ti_3c_ sites, the extraordinary reactivity of carboxylates with TiO_2_ surfaces is the dominant factor here.

**Figure 7:**
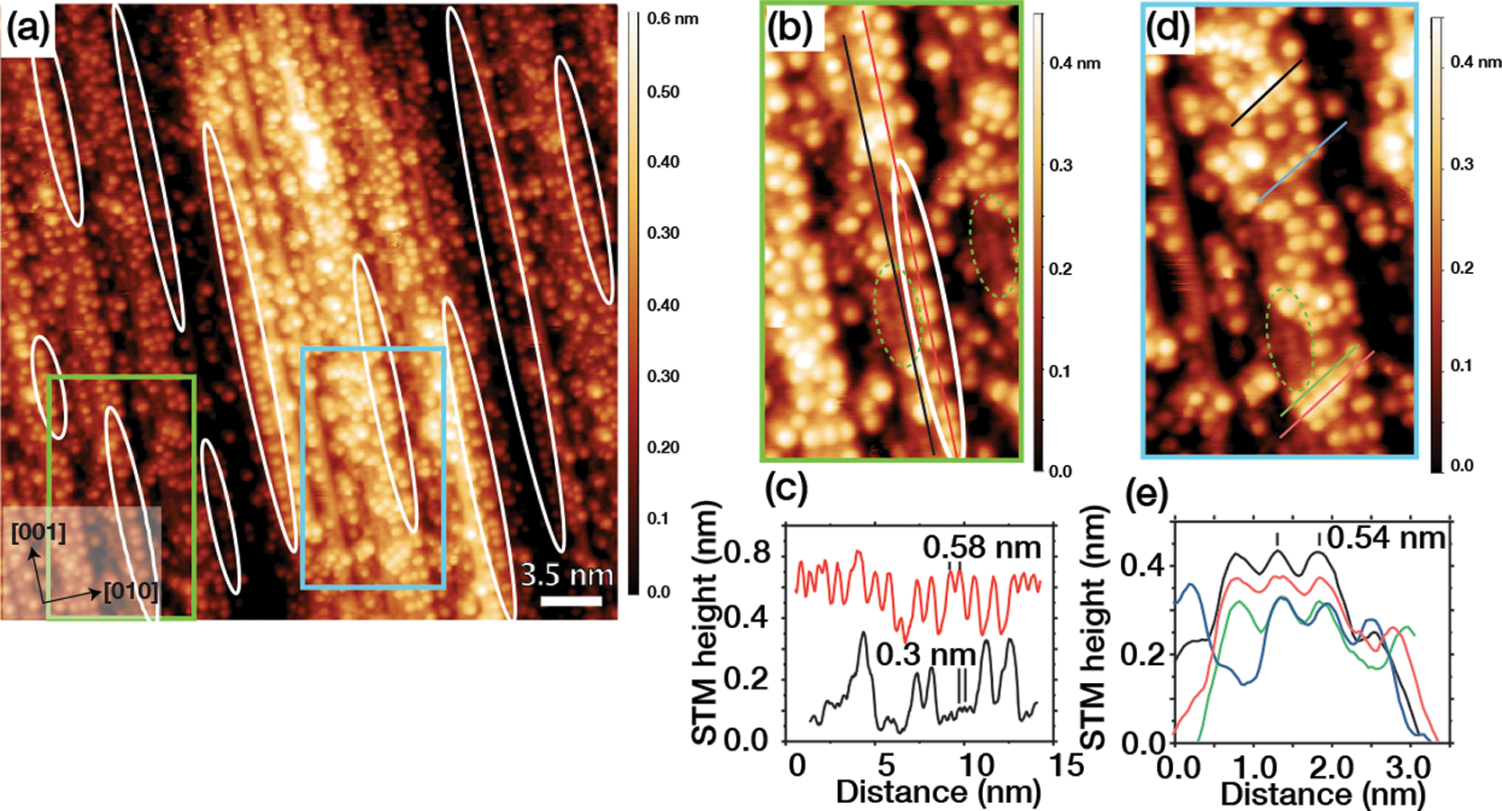
(a) 35 × 35 nm^2^ STM image of TiO_2_(100)-1 × 3 MF (*V*_s_ = +1.6 V, *I*_t_ = 0.1 nA) after exposure to 1.5 L trimethyl acetic acid. Solid white ovals depict quasi-ordering of trimethyl acetic acid along the [001] step edge rows. (b) 8 × 14 nm^2^ area of the image in (a) where line profiles (red, black) were obtained that are shown in (c). The green-dashed oval depicts an area where the periodicity of the bare substrate is observed. (c) Line profiles of the image in (b), which evidence adsorbate separations of 0.58 nm (red line) as well as a 0.3 nm 1× periodicity (black line). (d) 8.2 × 15.2 nm^2^ area of the image in (a), where molecules ordered in the diagonal direction are indicated. (e) Line profiles along the diagonal direction, which evidence an average spacing between adsorbates of 0.54 nm. The average minimum distance between adsorbates in (a) is 0.59 ± 0.09 nm.

Figure 7 shows STM images of TiO_2_(100)-1 × 3^MF^ after exposure to trimethyl acetic acid, which gives rise to an adsorbate coverage of 0.15 ± 0.04 ML. The stability of the overlayer again points to bidentate carboxylate adsorption. As for acetate on TiO_2_(100)-1 × 3^MF^, the overlayer is largely disordered with areas of ×2 order along step edges where steric hindrance effects are minimized. This is evidenced by a line profile separation of 0.58 nm. There is also one-dimensional ordering at 0.54 nm separation along the 57° diagonal seen for adsorption on the 1 × 1 surface (Figure 4). This could arise from restructuring of areas of 1 × 3^MF^ to 1 × 1, although the overall coverage is about half that obtained on the 1 × 1 surface (0.27 ML). For both carboxylates on TiO_2_(100)-1 × 3^MF^ the average minimum distance between adsorbates indicates that a greater coverage might be achieved by adsorption between existing domains. Indeed, in Figure 7 areas of the bare substrate are observed, with a line profile separation of 0.3 nm.

## Conclusions

Exposure of rutile TiO_2_ (100)-1 × 1 and the microfaceted (100)-1 × 3^MF^ reconstruction to acetic acid and trimethyl acetic acid results in dissociative adsorption. The STM results suggest that the resulting carboxylates are bidentate bonded to adjacent Ti_5c_ atoms along the [001] direction. A c(2 × 2) ordered overlayer of acetate is formed, which contrasts with the 2 × 1 overlayer formed on TiO_2_ (110). DFT calculations (using HSE06 functional; 25% Hartree–Fock) suggest that this difference arises from the increased Coulomb repulsion between adsorbates in the 2 × 1 overlayer on TiO_2_(100)-1 × 1 rather than relaxation or steric hindrance effects. Exposure of TiO_2_ (100)-1 × 1 to trimethyl acetic acid results in a largely disordered overlayer due to steric effects associated with the trimethyl group, although with ordering along the [001] rows at step edges where these effects are reduced. Steric hindrance also results in largely disordered adsorption of carboxylates on TiO_2_(100)-1 × 3^MF^, again with ordering at step edges. Since performance in applications such as DSSC will be modified by the degree of adsorbate order, these results provide design criteria for the nanoparticle supports.
